# Supplementary material for: Critical assessment of approaches for molecular docking to elucidate associations of HLA alleles with adverse drug reactions
Source: Mol Immunol. 2018 Sep;101:488–99. doi: 10.1016/j.molimm.2018.08.003 (PMC6148408; doi:10.1016/j.molimm.2018.08.003)
Supplement: Supplementary file 1 [file mmc1.docx]

**Supporting information**

**Supplementary Table 1. Percentage sequence identity between models and templates.**

|  | Template | |
| --- | --- | --- |
| Model | % Sequence Identity | |
| B*57:01 | B*52:01 | B*58:01 |
|  | 94% | 98% |
| B*57:03 | B*57:01 | B*07:02 |
|  | 99% | 91% |
| B*15:02 | B*15:01 |  |
|  | 99% |  |

Table to show the percentage sequence identity calculated using BLAST-P (Altschul et al, 1990). for each of the template sequences compared to the model sequences.


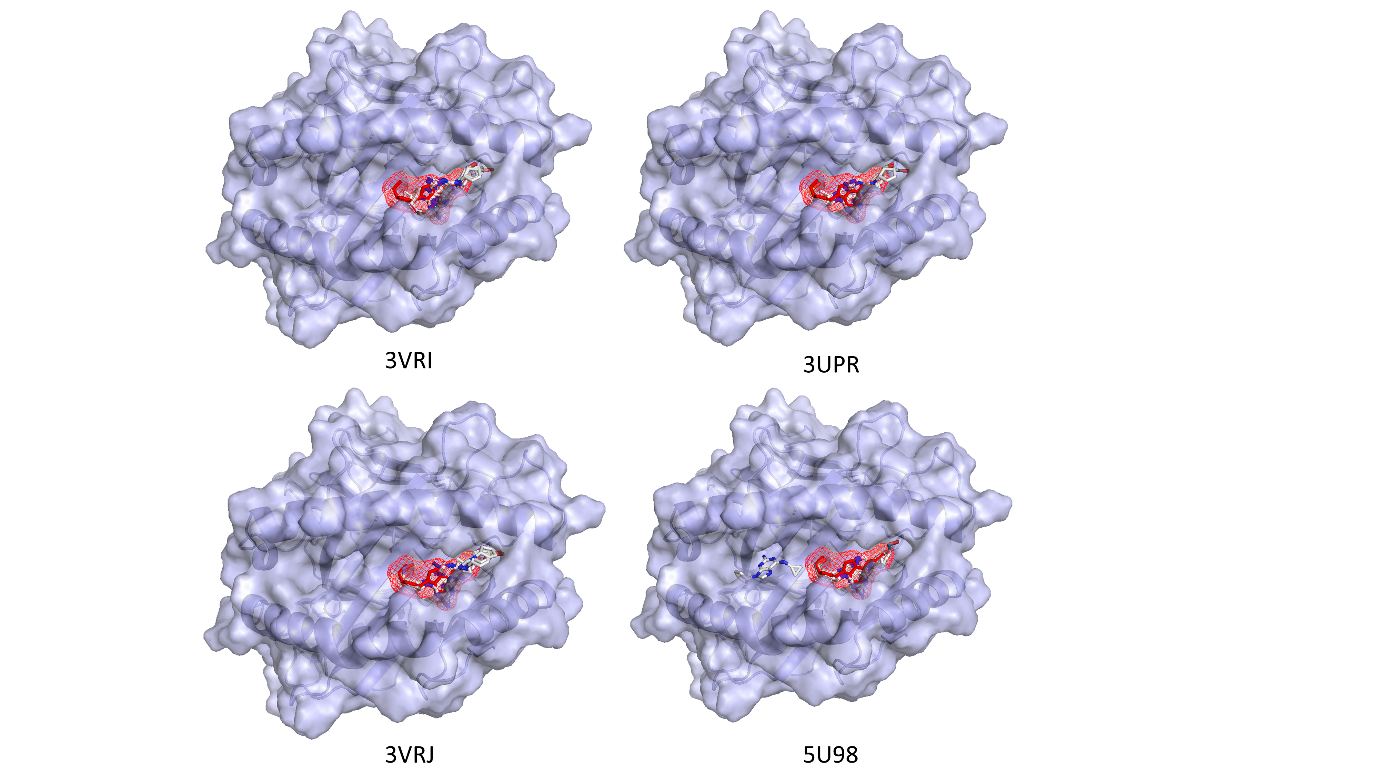
**Supplementary Fig 2. Comparison of B*5701 crystal structures.** Docking predictions of Abacavir with crystal structures of B*57:01 complexed with Abacavir and different peptides; 3VRI, 3UPR, 3VRJ and 5U98. Known binding position of Abacavir is shown in red. All structures show similar binding predictions.


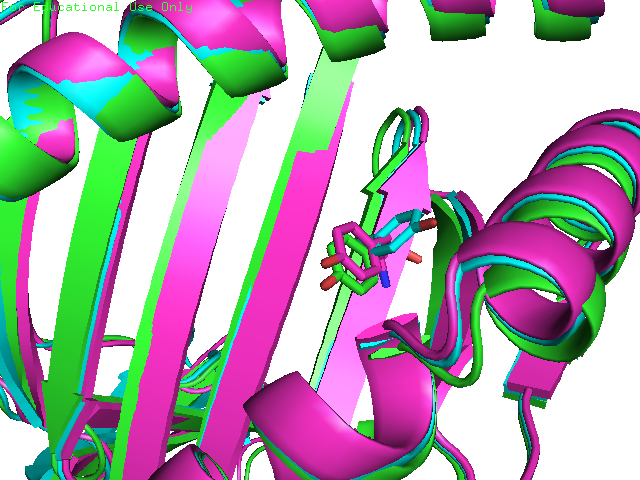

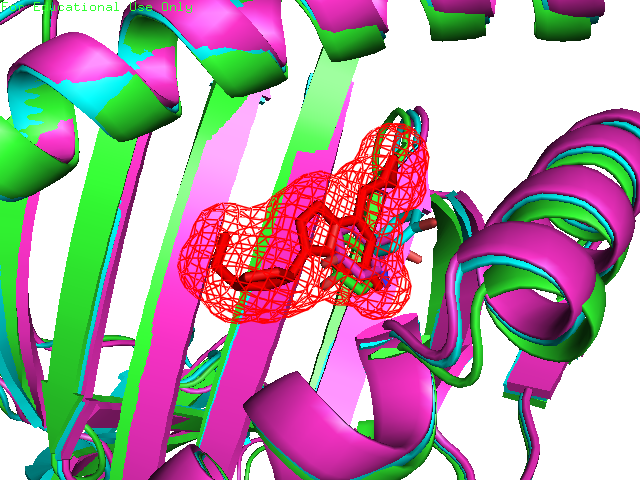


**Supplementary Fig 2. Crystal structure of B*57:03 aligned to modelled structure of B*57:03.** Crystal structure of B*57:03 (green) shown aligned with models created using one template (blue) and two templates (pink). Also shown with the known binding position of Abacavir (red). Looking at Tyr116 shown highlighted as sticks, it can be seen that the B*57:03 model created using one template shows a different conformation than expected by the known structure.


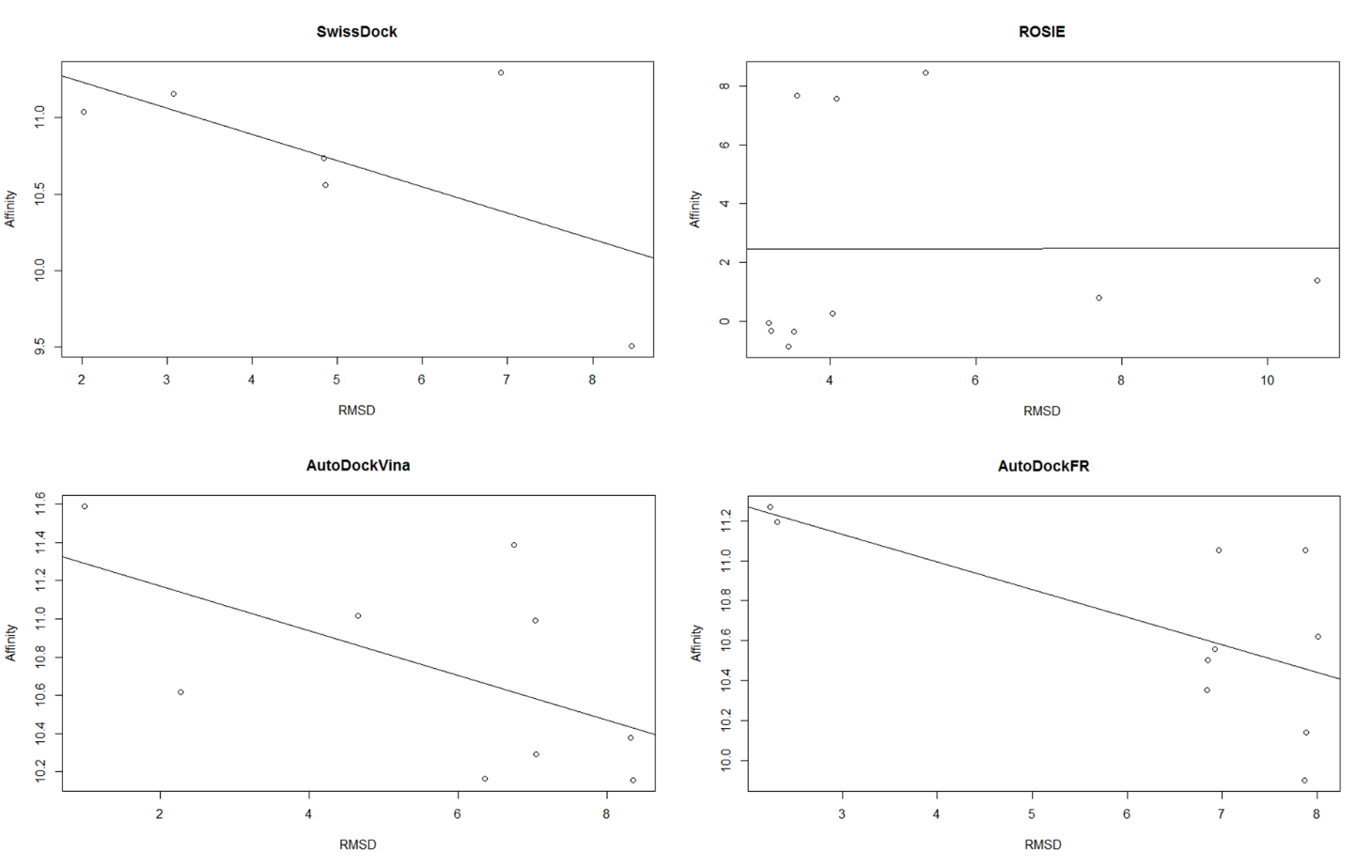


**Supplementary Fig 3. Scatterplots of Binding Affinity vs RMSD for Abacavir docking with B*57:01 using each of the software.** Scatter plots showing the relationship between RMSD and predicted binding affinity (-log10(KD|Ki)) for SwissDock (R^2^=-0.172), ROSIE (R^2^=0.00453), AutoDock Vina (R^2^=-0.117) and AutoDockFR (R^2^=-0.139).


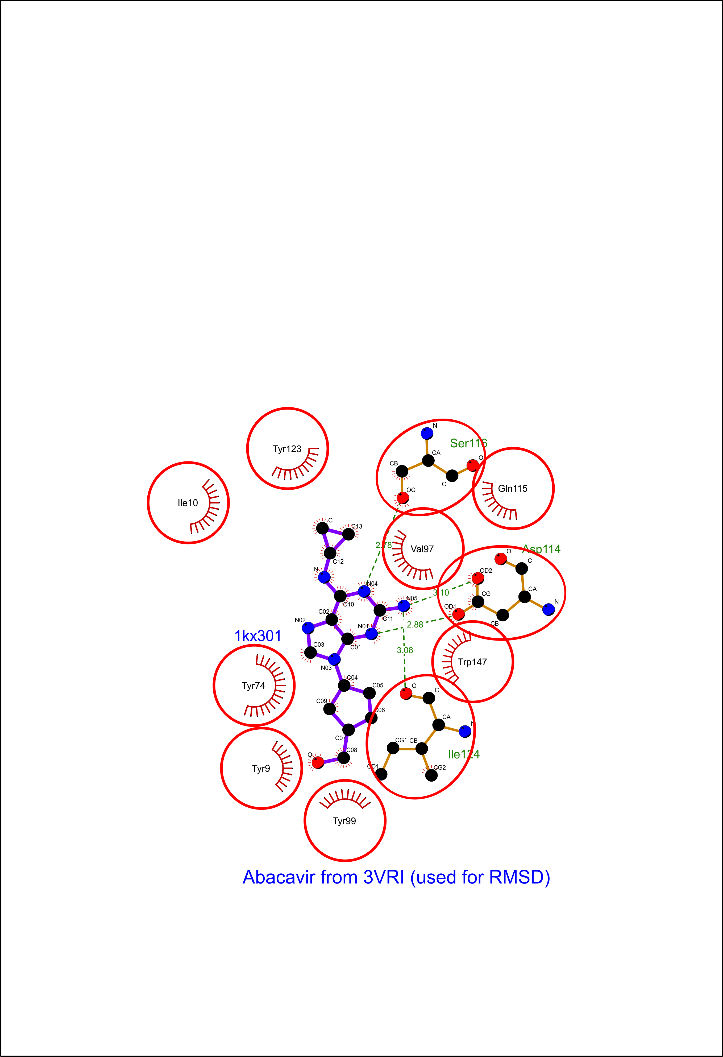

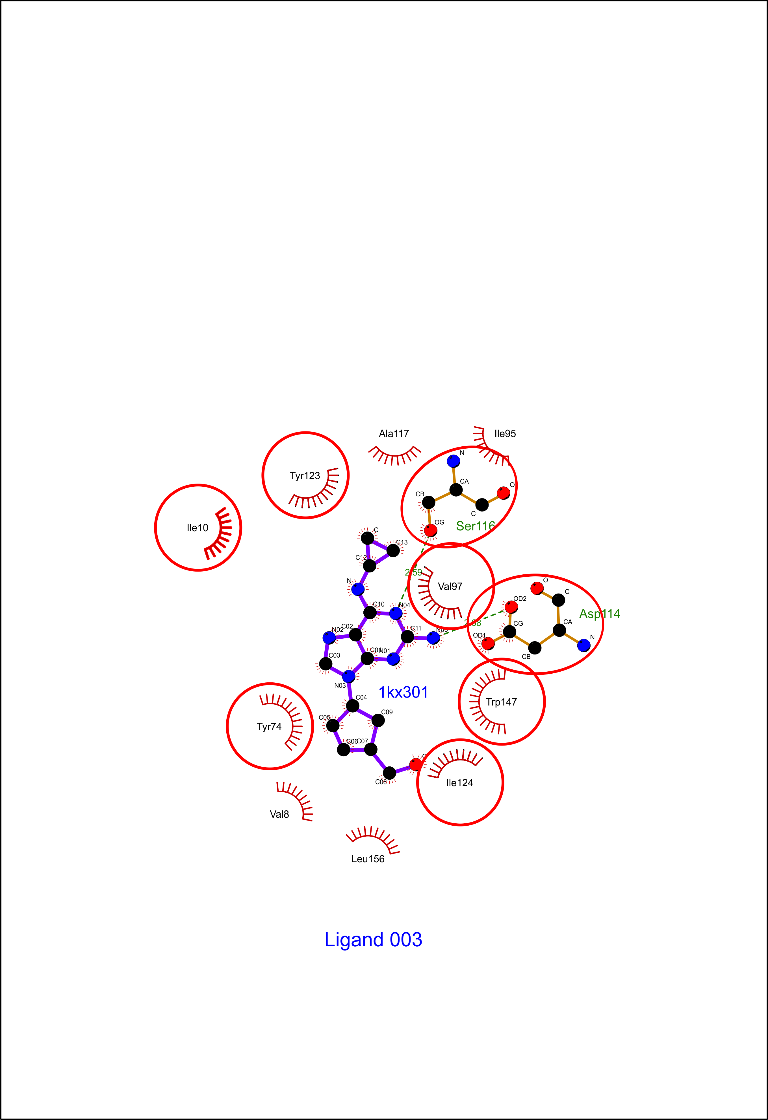

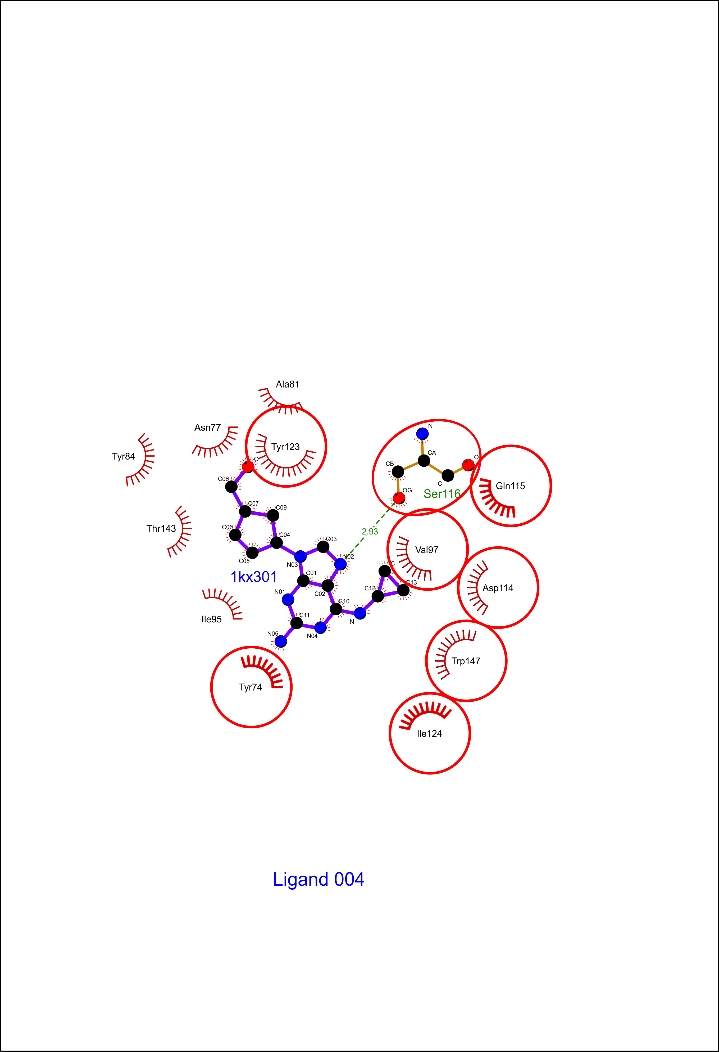


c)

b)

a)

**Supplementary Fig 4. Ligplot plots show the interactions between Abacavir and B*57:01.** (a) Known binding position of Abacavir in complex with B*57:01 (3VRI). (b) B5701_s using AutoDockFR, pose 3 showing lowest RMSD (2.254 Å). (c) B5701_s using AutoDockFR, pose 4 showing highest RMSD (7.639 Å). Similar interactions with key residues can be seen between all poses (circled). Dashed lines show Hydrogen bonds (with length), spoked arcs show hydrophobic bonds. Created using Ligplot (Wallace et al, 1996).


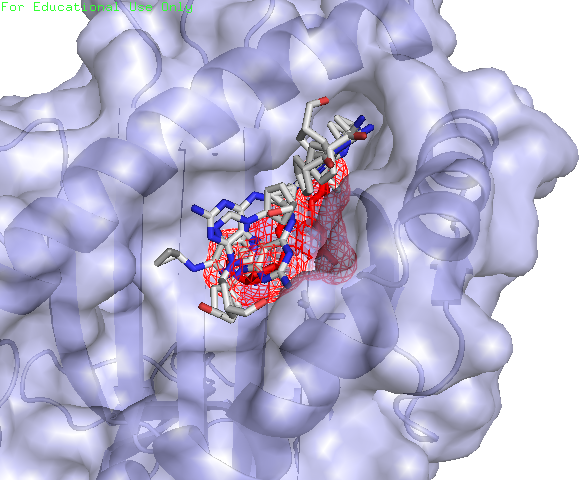


d)


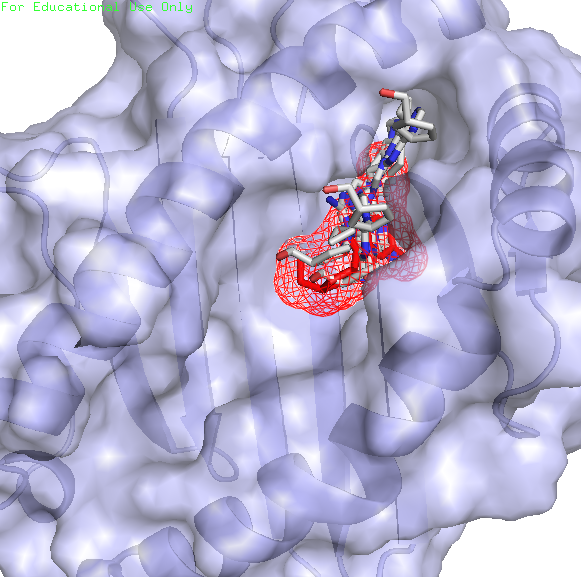


a)


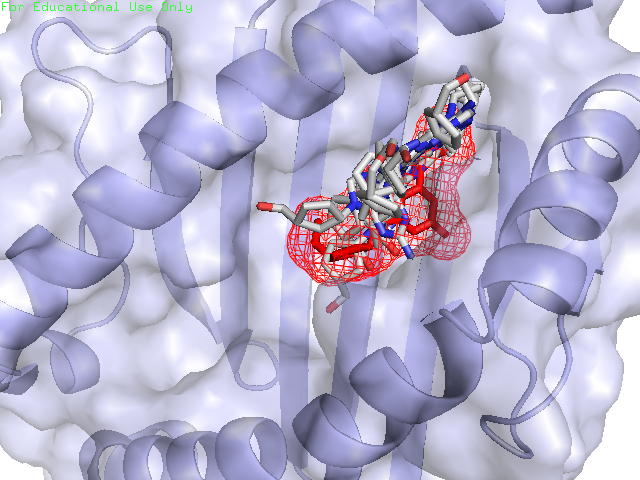


b)


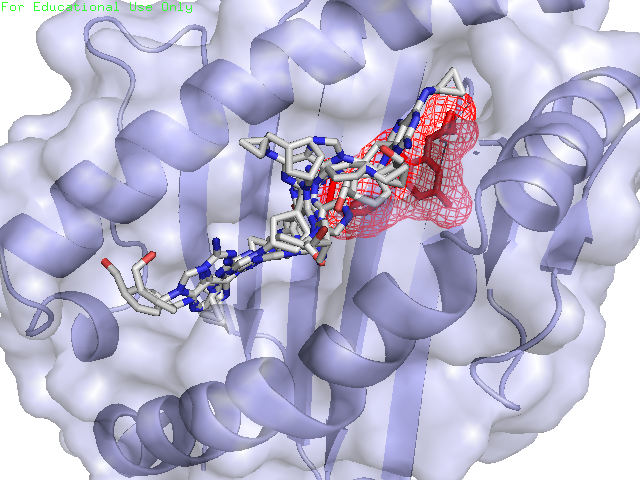


c)

**Supplementary Fig 5. Comparison of docking poses using crystal and modelled structures of B*57:01 and B*57:03.** (a) Known structure of B*57:01 (B5701_s) showing all docking poses for Abacavir using SwissDock; (b) Modelled structure of B*57:01 risk allele (B5701_m) showing all docking poses for Abacavir using SwissDock; (c); B*57:03 known structure (B5703_s) showing all docking poses for Abacavir using SwissDock; (d) modelled structure of B*57:03 (B5703_m2) showing all docking poses for Abacavir using SwissDock. Known binding position of Abacavir from 3VRI shown as red mesh.


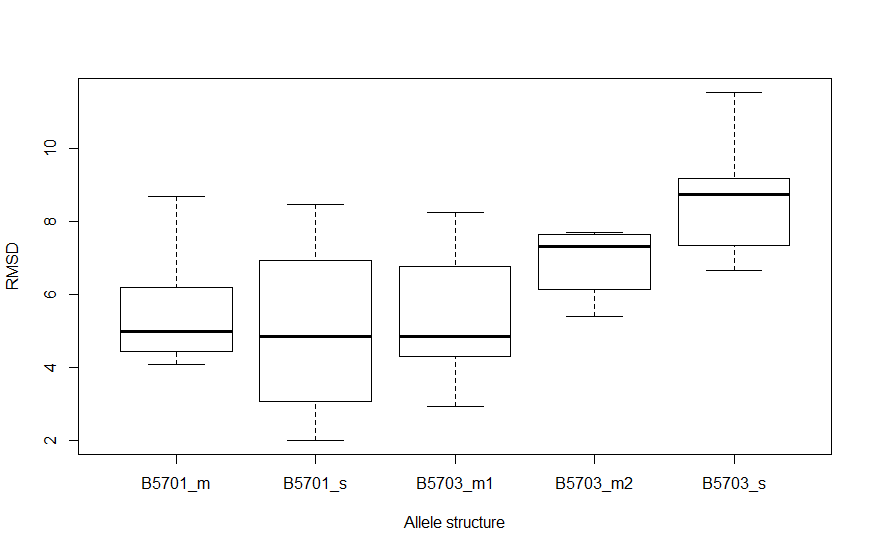


**Supplementary Fig 6. Comparison of RMSDs for docking poses using crystal and modelled structures of B*57:01 and B*57:03.** Boxplot to compare the RMSDs for poses compared to the known binding position of Abacavir, for both the crystal structures and models of B*57:01 and B*57:03 using SwissDock.

**
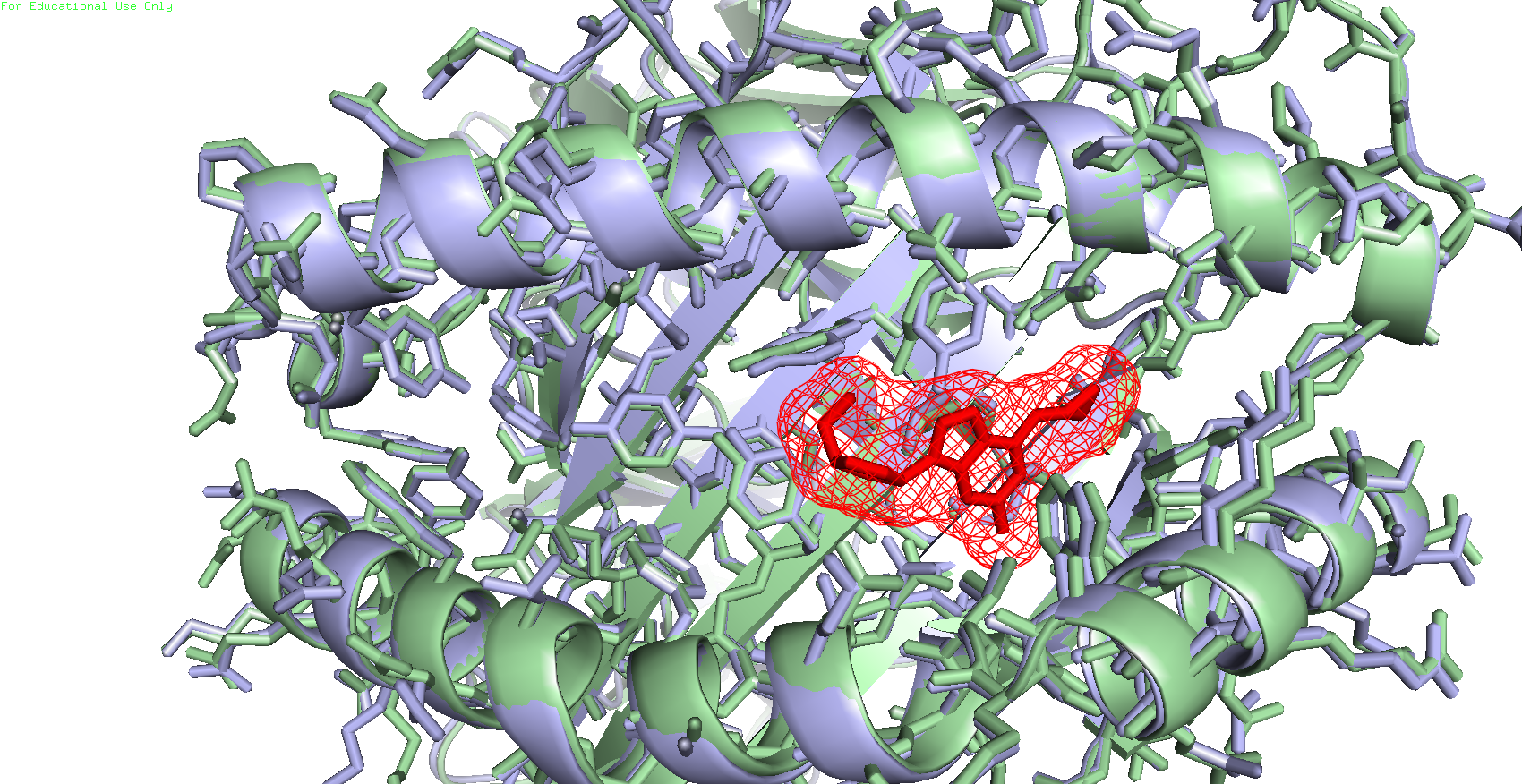
 Supplementary Fig 7. Comparison of B*57:01 crystal structure with only peptide bound (2RFX) with structure with Abacavir bound (3VRI).** Comparing the unbound structure of HLA-B*57:01, crystallised in the absence of drug (2RFX, shown in grey) with the bound structure (3VRI, shown in green). The known binding position of Abacavir is shown in red.


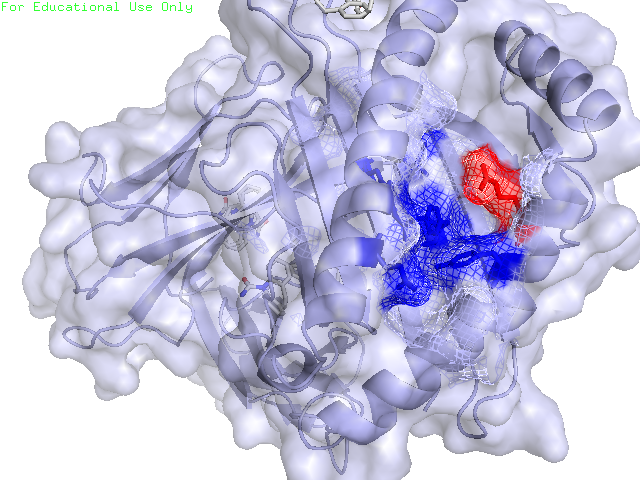

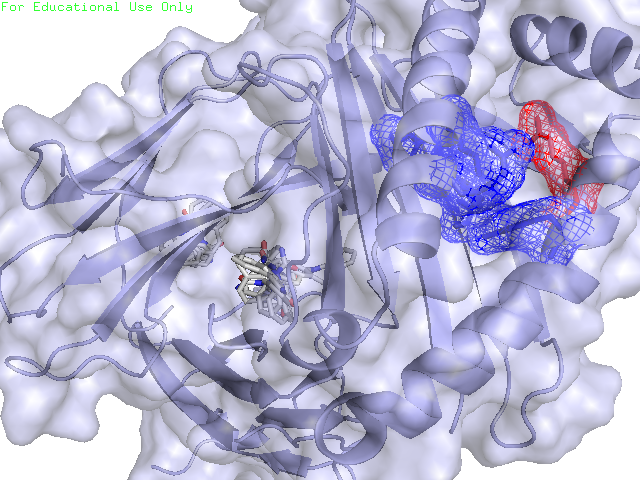

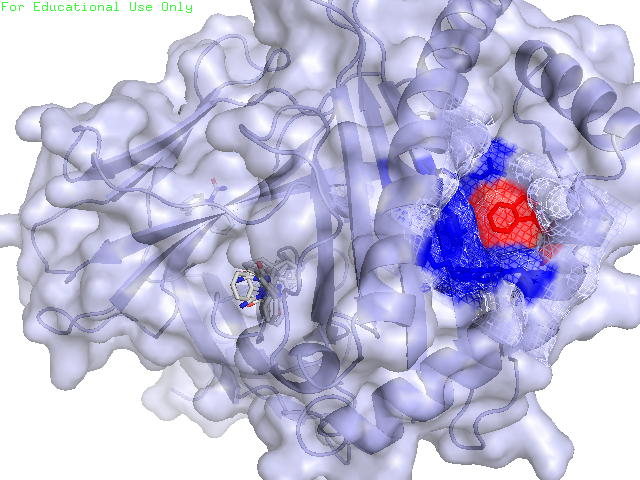


c)

b)

a)

**Arg156**

**Arg156**

**Trp156**

**Supplementary Fig 8. Docking Carbamazepine with control alleles using SwissDock.** SwissDock poses for Carbamazepine docked with (a) B*15:01 control allele (B1501_s), (b) B*07:02 control allele (B0702_s) and (c) A*01:01 control allele (A0101_s). Residue at 116 shown in red, with other D-pocket residues shown in blue.

References

Altschul S.F., Gish W., Miller W., Myers E.W. and Lipman D.J., Basic local alignment search tool, *J* *Mol. Biol.* **215**, 1990, 403–410.

Wallace A C, Laskowski R A, Thornton J M., LIGPLOT: a program to generate schematic diagrams of protein-ligand interactions. *Protein Eng.* **8**, 1996 127-134.
